# Invasive Functional Coronary Assessment in Myocardial Ischemia with Non-Obstructive Coronary Arteries: from Pathophysiological Mechanisms to Clinical Implications

**DOI:** 10.31083/j.rcm2311371

**Published:** 2022-10-31

**Authors:** Riccardo Rinaldi, Carmine Salzillo, Andrea Caffè, Rocco A. Montone

**Affiliations:** ^1^Department of Cardiovascular and Pulmonary Sciences, Catholic University of the Sacred Heart, 00168 Rome, Italy; ^2^Department of Cardiovascular Medicine, Fondazione Policlinico Universitario Agostino Gemelli IRCCS, 00168 Rome, Italy

**Keywords:** INOCA, MINOCA, pathophysiology, diagnosis, therapy

## Abstract

Despite ischemic heart disease (IHD) has been commonly identified as the consequence of obstructive coronary artery disease (OCAD), a significant percentage of patients undergoing coronary angiography because of signs and/or symptoms of myocardial ischemia do not have any significant coronary artery stenosis. Several mechanisms other than coronary atherosclerosis, including coronary microvascular dysfunction (CMD), coronary endothelial dysfunction and epicardial coronary vasospasm, can determine myocardial ischemia or even myocardial infarction in the absence of flow-limiting epicardial coronary stenosis, highlighting the need of performing adjunctive diagnostic tests at the time of coronary angiography to achieve a correct diagnosis. This review provides updated evidence of the pathophysiologic mechanisms of myocardial ischemia with non-obstructive coronary arteries, focusing on the diagnostic and therapeutic implications of performing a comprehensive invasive functional evaluation consisting of the assessment of both vasodilation and vasoconstriction disorders. Moreover, performing a comprehensive invasive functional assessment may have important prognostic and therapeutic implications both in patients presenting with myocardial ischemia with non-obstructive coronary arteries (INOCA) or myocardial infarction with non-obstructive coronary arteries (MINOCA), as the implementation of a tailored patient management demonstrated to improve patient’s symptoms and prognosis. However, given the limited knowledge of myocardial ischaemia with non-obstructive coronary arteries, there are no specific therapeutic interventions for these patients, and further research is warranted aiming to elucidate the underlying mechanisms and risk factors and to develop personalized forms of treatment.

## 1. Introduction

Ischemic heart disease (IHD) is the leading cause of disability and mortality worldwide and, traditionally, it has been identified with the presence of obstructive coronary artery disease (OCAD) (defined as any coronary artery stenosis >50%), with IHD and OCAD often used as interchangeable terms [1]. However, up to 50% of patients presenting with signs and/or symptoms of myocardial ischemia and undergoing coronary angiography do not have angiographic evidence of OCAD [2, 3, 4]. Several mechanisms other than coronary atherosclerosis, including coronary microvascular dysfunction (CMD), coronary endothelial dysfunction and epicardial coronary vasospasm, are implicated in myocardial ischemia or even myocardial infarction (MI) in the absence of angiographically evident flow-limiting epicardial stenosis. Therefore, to achieve a correct diagnosis there is the need of performing adjunctive diagnostic tests at the time of coronary angiography [5]. In addition, both CMD as well as epicardial coronary spasm have been associated with worse angina status as well as with up to 25% incidence of non-fatal MI, acute coronary syndromes, or hospitalization for heart failure and 5% incidence of all-cause mortality at follow-up [6, 7, 8, 9]. Furthermore, performing a comprehensive invasive functional assessment may have also important therapeutic implications both in patients presenting with myocardial ischemia with non-obstructive coronary arteries (INOCA) or myocardial infarction with non-obstructive coronary arteries (MINOCA) and, therefore, the catheterization laboratory represents the ideal opportunity to resolve diagnostic ambiguity, improve patients’ outcomes and optimize resource utilization [10, 11]. The aim of this review is to provide updated evidence of the pathophysiological mechanisms of myocardial ischemia with non-obstructive coronary arteries, focusing on the diagnostic and therapeutic implications of performing a comprehensive invasive functional evaluation in these patients. Indeed, gaining a deep insight in the underlying pathophysiologic mechanisms and the associated clinical implications could encourage cardiologists to perform additional test to achieve a final diagnosis as well as pave the way for further research and the development of novel therapeutic strategies.

## 2. Pathophysiologic Basis of Coronary Functional Assessment

Coronary arterial circulation can be described as two contiguous districts with different size and functions.

The proximal district is represented by the epicardial vessels, conductance arteries with cross-sectional diameter >500 μm visible on coronary angiography that normally contribute <10% to the total coronary vascular resistance. The pathogenetic mechanism responsible for myocardial ischemia in this compartment is conductance impairment, mostly caused by atherosclerotic plaque formation, intracoronary thrombus formation or epicardial coronary spasm [12]. Vasospastic angina (VSA) is the clinical manifestation of myocardial ischemia resulting from a dynamic epicardial coronary obstruction due to epicardial artery spasm. Vascular smooth muscle cells (VSMCs) hyper-reactivity is likely to be the key determinant of VSA, with several proposed mechanisms including an exaggerated Rho-kinase activation, an increased vagal or sympathetic activation, vascular and/or systemic inflammation, oxidative stress, personal exposures (e.g., smoking, air pollutants) and genetic predisposition [13, 14].

Conversely, the distal compartment is represented by the coronary microcirculation including all vessels with cross-sectional diameter <500 μm (i.e., pre-arteriolar vessels, intramural arterioles, and capillaries). This compartment is formed by resistive vessels and is responsible for >70% of the total coronary resistance, thus playing a central role in the physiological regulation of myocardial blood flow supply according to changes in the metabolic demands of the myocardium [15]. Indeed, the modulation of the vascular resistance achieved by the progressive vasodilation of coronary resistive arterioles can determine an increase of coronary blood flow (CBF) of up to five-fold in healthy individuals [16, 17]. CMD refers to the spectrum of functional and/or structural alterations of coronary microcirculation determining an impaired CBF and resulting in a supply-demand mismatch that may remain subclinical or cause symptomatic myocardial ischemia even in the absence of any epicardial stenosis [5, 18]. Microvascular angina (MVA) is the clinical manifestation of CMD and can result from structural remodelling of the microvasculature, such as adverse arteriolar remodelling, capillary rarefaction, and perivascular fibrosis, leading to a fixed reduced microcirculatory conductance due to impaired vasodilatation and/or increased microvascular resistance, and/or vasomotor disorders affecting the coronary arterioles and causing dynamic arteriolar obstruction due to microvascular spasm. Although the pathogenesis of CMD is not completely understood yet, oxidative stress together with a subsequent pro-inflammatory response are considered the main pathogenetic mechanisms. Reactive oxygen species accumulation may promote the over-production of vasoactive substances such as endothelin-1 and the reduction of nitric oxide (NO) production by endothelial cells, thus leading to an impaired NO-mediated vasodilatation. Furthermore, an increased RhoA/Rho-kinase activation in VSMCs has been associated with the increased tendency of coronary vessels to spasm [19, 20, 21]. The occurrence of CMD has been also proposed as a possible mechanism of myocardial injury (defined as cardiac troponin release) associated with severe acute respiratory syndrome coronavirus 2 infection [22].

## 3. Invasive Functional Coronary Assessment in Myocardial Ischemia with Non-Obstructive Coronary Arteries

Although coronary angiography remains the gold-standard for the diagnosis and exclusion of OCAD, the inability to directly visualize coronary microcirculation (beyond its resolution limit of 0.5 mm) substantially limits its diagnostic accuracy for coronary vascular disorders [23]. However, the filling of coronary vasculature by the angiographic contrast medium can still provide limited information regarding the function of coronary microcirculation. A retarded filling of the distal coronary vessels (the so-called coronary slow-flow) in the absence of OCAD has been used in previous studies as an indicator for CMD, with diagnostic criteria varying from a thrombolysis in myocardial infarction (TIMI) flow grade ≤2 or a corrected TIMI frame count >25 frames [24, 25].

**Fig. 1.**
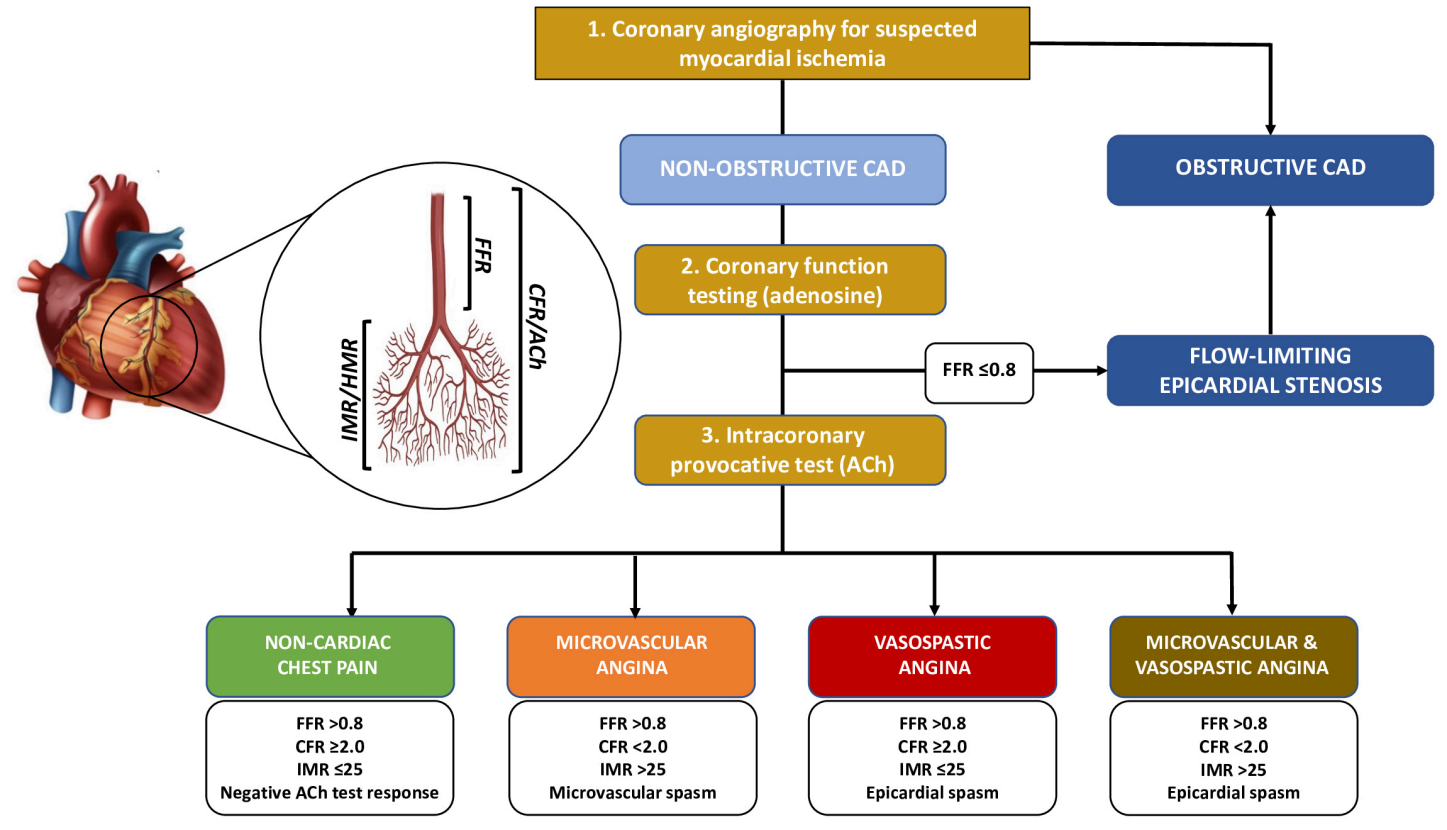
**Diagnostic algorithm for a comprehensive invasive functional assessment in patients with suspected myocardial ischaemia**. FFR, Fractional Flow Reserve; CFR, Coronary Flow Reserve; IMR, Index of Microvascular Resistance; HMR, Hyperaemic Microvascular Resistance; ACh, Acetylcholine; CAD, Coronary Artery Disease.

To overcome the limitations of coronary angiography, several invasive and non-invasive methods have been studied and validated to evaluate microvascular function. Despite the obvious limitations (besides low risks), the invasive approach remains the gold standard for the evaluation of the coronary artery vasculature offering several advantages such as the possibility to exclude haemodynamically significant epicardial CAD (i.e., by demonstrating a fractional flow reserve (FFR) value >0.80 in angiographically intermediate coronary stenosis, ranging from 40% to 90% according to European Society of Cardiology (ESC) Guidelines) [3] and to infuse intracoronary vasoactive agents to test coronary vasoreactivity in the same procedure. Moreover, a comprehensive invasive functional assessment consisting of coronary function testing, including coronary flow reserve (CFR) and microvascular resistance measurements, together with provocative test allows a combined assessment of the coronary microcirculation and the vasomotor function and the diagnostic distinction between impaired vasodilation of the microvasculature, increased microvascular resistance and increased vasoconstrictive response (Fig. 1) [9, 18, 25].

### 3.1 Coronary Function Testing for Microcirculation Assessment

The CFR is a physiological index that represents the vasodilator capacity of the entire coronary tree (i.e., both the epicardial vessels and the microvasculature) and evaluates the ratio of maximum hyperaemic to basal CBF and the extent to which myocardial coronary flow can increase above its baseline value in response to increased demand through the dynamical reduction of vascular resistance [5, 18, 26]. CBF can be estimated by measuring CBF velocity with an intracoronary Doppler flow wire (usually placed in the distal left anterior descending artery because of the large percentage of subtended myocardium and coronary dominance) or using a temperature sensor-tipped guidewire by measuring the saline bolus transit time at rest and in response to continuous infusion of pharmacological stressors (i.e., adenosine) to induce maximal hyperaemia with the thermodilution technique [27]. CFR can be impaired in patients with CMD and a defective hyperaemic microvascular dilatory response as well as in those with epicardial OCAD and, therefore, an impaired CFR (defined as a value <2.0) can be used for the diagnosis of impaired endothelium-independent function and CMD only once OCAD is ruled out [28]. An abnormal CFR has been associated with increased risk of mortality and cardiac events irrespective of the presence of OCAD and, moreover, the more severely impaired the CFR is, the higher the risk. In particular, Pepine *et al*. [6] reported that a lower CFR (<2.32) was associated with an increased risk for major adverse outcomes (death, nonfatal myocardial infarction, nonfatal stroke, or hospital stay for heart failure) in 189 women referred to evaluate suspected ischemia at a mean follow-up of 5.4 years. Likewise, AlBadri *et al*. [29] reported that a low CFR (<2.32) was an independent predictor of increased major adverse cardiovascular events (MACE) rate (defined as the composite of cardiovascular death, nonfatal MI, nonfatal stroke, or hospitalization for heart failure) in 224 in women without OCAD enrolled in the National Heart, Lung and Blood Institute-sponsored Women’s Ischemia Syndrome Evaluation (WISE) study at median follow-up of 9.7 years. Additionally, Lee *et al*. [30] showed that a low CFR (≤2) was associated with a higher rate of patient-oriented composite outcome (defined as any death, myocardial infarction, and revascularization) compared to those with high CFR in 313 patients with high FFR (>0.80) at median follow-up of 658 days. Finally, in a recent meta-analysis including 5,9740 subjects, Kelshiker *et al*. [31] demonstrated that an abnormal CFR was associated with a higher incidence of all-cause mortality (Hazard Ratio [HR]: 3.78, 95% confidence interval [CI] 2.39 to 5.97), a higher incidence of MACE (HR 3.42, 95% CI: 2.92 to 3.99), and each 0.1-unit reduction in CFR was associated with a proportional increase in mortality (per 0.1 CFR unit HR: 1.16, 95% CI: 1.04–1.29) and MACE (per 0.1 CFR unit HR: 1.08, 95% CI: 1.04–1.11).

However, some limitations of CFR must be acknowledged: first, it is an indirect estimation of true coronary flow; second, a steady-state hyperaemia with adenosine is required for its calculation, and its administration may cause adverse effects; third, thermodilution can overestimate CFR at higher values and is partially dependent on operator’s injections, thus determining a large intra-observer variability; finally, obtaining a stable signal during Doppler-based measurements may be challenging.

Recently, a novel method based on continuous thermodilution with stable hyperaemia achieved by intracoronary infusion of saline at room temperature has been introduced and validated to directly quantify absolute CBF and resistance, offering the potential advantages of being operator independent, highly reproducible, and not requiring adenosine administration. However, since normal values of absolute CBF and resistance are still a matter of debate, further research are needed before the implementation of absolute CBF measurement in daily clinical practice [32].

The index of microvascular resistances (IMR) is a dimensionless index that can be obtained by thermodilution as the product of the distal coronary pressure and mean transit time of a saline bolus during maximal hyperaemia induced by adenosine. Compared with CFR, IMR is independent on epicardial vascular function and hemodynamic conditions, thus providing a more reproducible assessment of the microcirculation [33, 34]. The cut-off of ≤25 is currently used for “normal” and >25 for increased microvascular resistance. A combined evaluation with CFR and IMR can provide a more comprehensive evaluation of the microcirculatory function given that, due to coronary autoregulation, CFR can still be within normal values even in the presence of increased microvascular resistance [35]. Alternatively, the hyperaemic microvascular resistance (HMR) uses Doppler flow velocity to estimate flow instead of thermodilution. HMR is measured in mmHg/cm/s and values >2.5 are considered abnormal. However, the role of HMR is less well established yet, as most studies mainly focused on the thermodilution-derived parameters (CFR and IMR).

### 3.2 Intracoronary Provocative Tests

The diagnosis of coronary vasomotor disorders is usually achieved by performing an intracoronary provocative test with pharmacologic vasoactive agents that can trigger a vasoconstrictive response at the epicardial and/or microvascular level in susceptible individuals [36, 37].

The most used vasoactive provocative agent to explore endothelium-dependent vasodilation in clinical practice is acetylcholine (ACh): given that ACh indirectly induces vasodilation by stimulating the release of NO from endothelial cells and directly promote VSMCs contraction, the resulting effect of intracoronary ACh administration can be either vasodilatation (healthy endothelium) or coronary spasm (endothelial dysfunction or increased VSMCs reactivity) [38]. The safety of intracoronary provocative tests with ACh has been widely investigated both in patients with INOCA and MINOCA with a relatively low risk of transient complications (mainly represented by transient bradyarrhythmia and supraventricular tachycardia) and performing a provocative test may have relevant prognostic implications, as a positive ACh test (either for microvascular or epicardial spasm) is associated with a higher risk of future cardiovascular events [9]. In particular, a recent study demonstrated that ACh provocation testing is associated with a low risk of mild and transient complications (mainly represented by transient bradyarrhythmia) but patients with a positive test had a higher incidence of major adverse cardiovascular and cerebrovascular events (defined as the composite of cardiovascular death, nonfatal MI, hospitalisation due to unstable angina, and stroke/transient ischaemic attack) compared to those with a negative result at a median follow-up time of 22 months [39].

As an alternative to ACh, ergonovine (ER) can be used as vasoactive agent for intracoronary provocative test. ER mainly acts through serotonergic receptors (5-HT2) on VSMCs, inducing an endothelium-independent vasoconstriction in susceptible vessels. Moreover, ER may also cause the release of relaxing prostanoids from the healthy endothelium, a process that can be compromised in the presence of endothelial dysfunction, in which ER favours vasoconstriction [40].

Epicardial spasm is diagnosed when the typical ischemic symptoms (i.e., chest pain) are reproduced by ACh infusion in association with ischemic electrocardiographic (ECG) changes (e.g., ST segment depression or elevation) and coronary artery spasm defined as transient total or subtotal coronary artery occlusion (≥90% diameter reduction from baseline) in any epicardial segment [13]. Microvascular spasm is diagnosed when the typical ischemic symptoms and ECG changes are reproduced by ACh without evidence of epicardial coronary spasm (<90%) [25]. An intracoronary Doppler flow wire can be used to continuously measure CBF during the provocative test and, in the absence of any significant epicardial spasm, the occurrence of microvascular spasm will cause a reduction in CBF and could be diagnosed by an ACh flow reserve (CBF during ACh infusion/CBF at rest) less than 1. As the alterations in CBF usually occur earlier in the ischemic cascade than ECG changes and symptoms, the continuous measurement of CBF during the provocative test could allow an earlier and more sensitive diagnosis of coronary microvascular spasm [27].

## 4. Clinical Implications of Invasive Functional Assessment

### 4.1 Invasive Functional Assessment in INOCA

The term INOCA identifies a significant proportion of patients (up to 50% and particularly women) referred for coronary angiography because of stable, chronic ischaemic symptoms (stable angina or angina equivalent) and/or signs of ischemia on non-invasive testing (i.e., exercise stress test, stress echocardiography or nuclear imaging) and found to have normal or near-normal coronary arteries [3, 5].

In the most recent ESC guidelines, performing an invasive coronary function testing for measurement of CFR and IMR/HMR have a Class IIa (“should be considered”) recommendation in patients with INOCA. On the contrary, performing an intracoronary provocative test with ACh have a Class IIb recommendation (“may be considered”) for the diagnosis of MVA and a Class IIa if VSA is suspected [3]. However, the rationale for performing adjunctive functional tests in these patients is strong and threefold: diagnosis, treatment and prognostic implications. Indeed, a comprehensive functional assessment, including both coronary function testing and provocative test, allows the identification of specific endotypes within the heterogeneous population of INOCA characterized by distinct mechanisms and/or responses to medical therapy (i.e., MVA, VSA, both MVA and VSA, or none) (Table 1) [36]. The “Coronary Microvascular Angina” (CorMicA) trial demonstrated that performing an invasive functional assessment in INOCA patients and implementing a consequent tailored medical therapy are associated with significant improvements in patients’ outcomes in terms of reduction of angina severity and quality of life [41]. Indeed, if the specific endotype is not correctly identified nor an appropriated medical therapy is not instituted, INOCA patients may often experience recurrent angina, with hospital readmission and repeated invasive procedures. This is particularly relevant, as INOCA patients are usually younger than patients with OCAD [36]. Therefore, in patients with signs and/or symptoms of myocardial ischemia without angiographic evidence of OCAD, coronary angiography should be considered incomplete without adjunctive diagnostic tests aiming to assess the presence of coronary vascular dysfunction.

**Table 1.** **Classification of INOCA in four endotypes according to the results of coronary function testing and the response to intracoronary provocative test with ACh**.

(A) Microvascular Angina	(B) Vasospastic Angina	(C) Both Microvascular and Vasospastic angina	(D) None/non-cardiac chest pain
Evidence of CMD defined as any of:	Normal CFR (≥2.0)	Evidence of CMD and epicardial spasm	Normal CFR (≥2.0)
	Normal IMR (<25)		Normal IMR (<25)
Abnormal CFR (<2.0)	Epicardial spasm		Neither microvascular nor epicardial spasm
Abnormal IMR (≥25)			
Microvascular spasm			

INOCA, Ischemia with Non-Obstructive Coronary Arteries; ACh, Acetylcholine; CMD, Coronary Microvascular Dysfunction; CFR, Coronary Flow Reserve; IMR, Index of Microvascular Resistance.

Treatment of INOCA patients should target the underlying risk factors and pathogenic mechanism of the different endotypes. However, to date, there are no disease-modifying therapies specific to INOCA, and there is a strong need for further research to address this unmet clinical need.

Traditional cardiovascular risk factors, such as hypertension, dyslipidaemia, smoke habit, and diabetes, are all relevant contributor to the development of coronary microvascular and vasospastic dysfunction as well as to determine a structural remodelling of the coronary circulation. In particular, hypertension has been strongly associated with the adverse remodelling of coronary microvasculature and, therefore, an optimal control of blood pressure is fundamental to prevent the progression of CMD and obtain a reduction in angina frequency and intensity [36]. The choice of the best medications should be based upon the predominant endotype (e.g., VSA, MVA or both).

Standard anti-ischemic medications often obtained disappointing results in patients with INOCA. Long-acting nitrates may help to reduce angina episodes, but their efficacy in reducing MACE and improving CMD was not demonstrated [42]. Furthermore, they may worsen anginal symptoms in MVA due to a stealing effect [43]. Short-acting nitrates, although useful to treat acute anginal attacks especially if an abnormal vasodilator reserve is present, are usually only partially effective [20]. Calcium-channel blockers (CCBs) are particularly effective in presence of both microvascular and epicardial spams, and most experts’ consensus indicate CCBs as the first-line agents when the presence of vasomotor disorders is either suspected or documented [36]. In particular, CCBs demonstrated to improve angina status and reduce the rate of MACE in patients with VSA [44].

If MVA is associated with an abnormal CFR and/or an increased IMR, thus suggesting the presence of adverse arterial remodelling, β-blockers, CCBs, and angiotensin converting enzyme inhibitors (ACEi) could be beneficial [5]. In particular, ACEi demonstrated to restore endothelial function and improve hyperaemic CBF in patients with hypertension and MVA as well as to improve CFR and reduce anginal symptoms in women with CMD [44, 45]. The same beneficial effects have been reported also with angiotensin-II receptor blockers (ARBs) [45].

In patients with MVA and effort-induced angina with evidence of increased adrenergic activity, β-blockers demonstrated to improve anginal symptoms and, therefore, they should be the first line therapy in these patients [21]. However, β-blockers may worsen the occurrence of epicardial spasm and should be avoided in these patients as they may promote coronary vasoconstriction by unmasking α-adrenoreceptors in the coronary circulation [46].

Statins demonstrated to reduce angina recurrence and the rate of MACE in patients with epicardial spasm as well as to improve endothelial dysfunction and CFR in patients with CMD, probably due to their anti-inflammatory and anti-oxidant properties [47].

Nicorandil, a vasodilator drug that induces the relaxation of coronary VSMCs by stimulating guanylyl cyclase and increasing cyclic guanosine monophosphate (cGMP) levels as well as by inducing the activation of K+ channels and hyperpolarization, was proven to prevent exercise induced myocardial ischemia (i.e., both time to 1-mm ST depression and total exercise duration at treadmill exercise test) in patients with CMD without modifying the heart rate variability, thus suggesting a direct vasodilatory effect on coronary microvasculature [48].

Ranolazine, an inhibitor of the late inward sodium current that enhances myocyte relaxation and ventricular compliance by reducing intracellular calcium levels, improved anginal symptoms and myocardial perfusion reserve in patients with MVA and a severely reduced CFR owing to an impaired vasodilation [49].

Ivabradine, a heart-rate-lowering agent that acts by selectively inhibiting the cardiac pacemaker current (If), could likely improve persistent anginal symptoms in selected patients, but its role in MVA is still controversial and barely investigated [50].

Fasudil is a selective Rho-kinase inhibitor that induces vasodilation by reducing the phosphorylation of the myosin light chain phosphatase, thus increasing phosphatase activity and preventing VSMCs contraction. Recent evidence demonstrated its efficacy in preventing coronary spasm and myocardial ischemia in patients with evidence of epicardial and/or microvascular spasm as well as to reduce microvascular resistances in patients with increased IMR [51, 52, 53].

Finally, Zibotentan is a potent and selective oral antagonist of endothelin A receptors that could be beneficial by contrasting the increased vasoconstrictive response of coronary microcirculation to endothelin in patients with MVA. To this aim, the ongoing Precision Medicine With Zibotentan in Microvascular Angina (PRIZE) randomized controlled trial (NCT04097314) will evaluate whether the add-on treatment with Zibotentan could improve treadmill exercise times in patients with MVA and impaired exercise intolerance [54].

The Women’s IschemiA TRial to Reduce Events In Non-ObstRuctive CAD (WARRIOR) is a multicenter, prospective, randomized, blinded outcome trial evaluating a strategy of intensive medical therapy compared with usual care in 4422 symptomatic women with INOCA (NCT03417388). The hypothesis is that an intensive medical therapy consisting of high-intensity statin, maximally tolerated ACEi/ARBs, and aspirin could reduce the primary outcome of first occurrence of MACE defined as the composite of all-cause death, non-fatal MI, non-fatal stroke, or hospitalization for chest pain or heart failure by 20% compared to usual care consisting of symptom control and primary risk reduction at ∼2.5-year follow-up [55].

Moreover, it is important to highlight the prognostic implications of performing an invasive functional assessment, as INOCA patients are at increased risk for future cardiovascular events (including acute coronary syndromes, heart failure hospitalization, stroke and repeated cardiovascular procedures) [56]. Upon the results of invasive coronary function testing, patients with CMD may be further classified according to IMR into distinct ‘structural’ (low CFR, high IMR/HMR) and ‘functional’ (low CFR, normal IMR/HMR) endotypes, with distinct underlying pathophysiological process that could represent therapeutic targets in the future. The ‘structural CMD’ endotypes are more frequently associated with an increased risk of acute coronary syndromes and mortality, while ‘functional CMD’ is associated with an increased risk of hospitalizations for recurrent angina [57].

Finally, even in patients with clear angiographic evidence of OCAD, the presence of coexistent coronary microvascular abnormalities both at epicardial and/or microvascular level may determine myocardial ischemia in territories supplied by healthy coronary arteries as well as contribute to reduce CFR and may worsen myocardial ischemia in territories supplied by arteries with significant CBF reduction due to the presence of epicardial coronary stenosis. These mechanisms may partially justify the results of the recent International Study of Comparative Health Effectiveness with Medical and Invasive Approaches (ISCHEMIA) trial demonstrating the limited prognostic efficacy of revascularization in subjects with inducible moderate-to-severe ischemia and ≥50% stenosis in a major epicardial vessel and highlight the importance of properly investigate the microvascular compartment and consider mechanisms independent of OCAD in determining ischemic symptoms in all patients [58].

### 4.2 Invasive Functional Assessment of MINOCA

MINOCA accounts for up to 6–8% of patients presenting with acute MI and is defined as the evidence of MI with normal or near-normal coronary arteries at coronary angiography without any alternative diagnosis for clinical presentation (e.g., sepsis, pulmonary embolism, tachyarrhythmias, myocarditis and Takotsubo syndrome). A variety of pathogenetic mechanisms may result in MINOCA (i.e., coronary plaque rupture/erosion, spontaneous coronary artery dissection, epicardial/microvascular spasm, and coronary embolism) and MINOCA should be considered a heterogeneous working diagnosis requiring a comprehensive assessment aiming to investigate the potential underlying aetiologies [59, 60, 61].

Despite some initial concerns, it has been widely demonstrated that performing an intracoronary provocative test with ACh for coronary vasomotor evaluation in the acute phase is safe and allows the detection of coronary vasoconstriction disorders at either epicardial or microvascular level as well as the start of a tailored medical therapy. Moreover, the presence of coronary functional alterations has also prognostic implications, as a positive ACh test (either at epicardial or microvascular level) identifies a high-risk subset of patients with an increased risk of future cardiovascular events [9, 10, 38]. To date, the management of MINOCA is still scarcely supported by evidence-based literature and current guidelines do not specifically provide recommendations regarding acute and long-term management of MINOCA (Table 2).

**Table 2.** **Therapeutic and prognostic implications of invasive functional assessment in myocardial ischemia and non-obstructive coronary arteries according to clinical presentation**.

Clinical presentation	Therapeutic implication	Prognostic implications
INOCA	Identification of specific endotypes (MVA, VSA, both MVA and VSA, none) and the start of a tailored medical therapy according to the different endotypes	A tailored medical therapy based upon the specific underlying mechanism of INOCA demonstrated to improve clinical outcomes.
	Consider ACEi and statins in all patients	Patients with CMD may be classified according to IMR into ‘structural’ (low CFR, high IMR/HMR) and ‘functional’ (low CFR, normal IMR/HMR) endotypes.
VSA	CCBs (1st-line agents, demonstrated to improve angina status and reduce the rate of MACE)	Higher risk of MACE, especially within 3 months of symptoms onset or even in asymptomatic patients.
	Long-acting nitrates (no efficacy in reducing MACE and improving CMD)	Smoking cessation and CCBs therapy are the most determinant prognostic factors.
	Nicorandil	IMR >18U has been associated with a higher occurrence of MACE.
	Fasudil (especially if increased IMR)	
	Avoid β-blockers	
MVA Microvascular spasm	CCBs	Generally better prognosis compared with epicardial spasm.
	Nicorandil	
	Fasudil (especially if increased IMR)	
	Nitrates may aggravate symptoms due to a stealing effect.	
MVA Functional CMD	β-blockers (especially if effort-induced angina)	More frequently associated with chest pain hospitalizations.
MVA Structural CMD	CCBs	More frequently associated with acute coronary syndromes and deaths.
	Nicorandil	
	Ranolazine (especially if markedly reduced CFR)	
	Ivabradine	
VSA and MVA Mixed type	CCBs	Particularly high risk of MACE.
	Nicorandil	
	Fasudil	
MINOCA	Performing an invasive provocative test for coronary vasomotor evaluation allow the detection of coronary vasoconstriction disorders and the start of a tailored medical therapy.	MINOCA patients with a positive intracoronary provocative test (i.e., epicardial or microvascular spasm) are at higher risk for future cardiovascular events.
	The presence of myocardial bridge should induce to perform an invasive provocative test in MINOCA patients. If positive, avoid β-blockers and use CCBs.	A positive intracoronary provocative test in patients with myocardial bridge and MINOCA is associated with a worse medium-long term outcome.

INOCA, Ischemia with Non-Obstructive Coronary Arteries; MVA, Microvascular Angina; VSA, Vasospastic Angina; ACEi, Angiotensin Converting Enzyme inhibitors; CMD, Coronary Microvascular Dysfunction; CFR, Coronary Flow Reserve; IMR, Index of Microvascular Resistance; HMR, Hyperaemic Microvascular Resistance; CCBs, Calcium Channel Blockers; MACE, Major Adverse Cardiovascular Events; MINOCA, Myocardial Infarction with Non-Obstructive Coronary Arteries.

Moreover, the effects of secondary preventive treatments beneficial in myocardial infarction due to OCAD are still largely unknown in MINOCA, with few prospective trials exploring this fields [62]. In particular, the ongoing “Randomized Evaluation of Beta Blocker and ACEi/ARBs treatment of MINOCA patients” (MINOCA-BAT) clinical trial aims to determine whether β-blockers and/or ACEi/ARBs may reduce the composite endpoint of all-cause mortality, readmission for MI, ischemic stroke or heart failure in MINOCA (NCT03686696) [63]. Similarly, the ongoing “Stratified Medicine of Eplerenone in Acute MI/Injury” (StratMed-MINOCA) clinical trial aims to evaluate if a stratified medicine approach with early risk stratification by CMD (defined as an IMR ≥25) coupled with mineralocorticoid antagonist therapy (i.e., eplerenone) may limit myocardial damage defined as changes in N-terminal prohormone of brain natriuretic peptide (NCT05198791). Furthermore, the SWEDEHEART registry demonstrated a significant reduction of cardiovascular events in MINOCA patients treated with statins and ACEi/ARBs, a trend for a beneficial effect of β-blockers but, of note, the use of dual antiplatelet therapy (DAPT) showed no prognostic benefits. However, the heterogeneous nature of the MINOCA cohort without discerning the underlying pathogenetic mechanisms represents an important limitation of this study [64, 65]. Indeed, it is still unknow whether a personalized approach based on the underlying pathophysiological mechanism and a consequent tailored therapy could be beneficial in these patients [66, 67]. For this purpose, the ongoing “PROgnostic value of precision medicine in patients with Myocardial Infarction and non-obStructive coronary artEries” (PROMISE) trial (NCT05122780) will evaluate whether a precision medicine approach with adjunctive diagnostic tests aiming to nvestigate the underlying pathophysiological mechanism (i.e., optical coherence tomography to assess plaque rupture or plaque erosion, intracoronary ACh provocative test to assess coronary vasomotor disorders, transoesophageal echocardiography and/or contrast enhanced echocardiography if distal/microvascular embolization is suspect, and cardiac magnetic resonance) and a consequent tailored pharmacological approach could be superior to a standard approach of coronary angiography alone and standard therapy for MI (DAPT in all patients, β-blockers, statins and ACEi/ARBs if clinically indicated) in terms of angina reduction and better quality of life at follow-up in MINOCA [68].

### 4.3 Invasive Functional Assessment in Patients with Myocardial Bridging

Mocardial bridging (MB) is a congenital coronary anomaly in which a segment of an epicardial coronary artery extends intramurally through the myocardium for a portion of its length below a muscular bridge. The prominent angiographic finding revealing the presence of MB is the dynamic compression during systole of the involved epicardial coronary artery [69].

Even if initially considered a benign condition, recent evidence showed that patients with MB without angiographic evidence of OCAD undergoing intracoronary provocative test with ACh may frequently present endothelial dysfunction either at epicardial or microvascular level. Moreover, the presence of coronary vasomotor disorders in these patients is an important yet often overlooked cause of MINOCA [70]. Therefore, the presence of MB should hint to perform an intracoronary provocative test with ACh, particularly in patients presenting with an acute clinical presentation. Indeed, a positive result in these patients has relevant prognostic implications as it has been associated with an increased rate of MACE at follow-up. Finally, a positive provocative test result in patients with MB has also therapeutic implications, as β-blockers and CCBs represent the first-line medical therapy for MB. However, given that β-blockers may favour the occurrence of coronary spasm, performing an intracoronary provocative test with ACh may be useful to guide a tailored management of these patients, with the introduction of CCBs rather than β-blockers if evidence of vasospasm [71].

## 5. Conclusions and Future Directions

This review demonstrates that performing a comprehensive invasive functional assessment consisting of the assessment of both vasodilation and vasoconstriction disorders at the time of coronary angiography is important for the decision making in patients with IHD as it allows to evaluate the whole coronary vascular tree from epicardial vessels to coronary microcirculation and to establish a correct diagnosis. Moreover, this review provides evidence that, even in the absence of OCAD, the presence of coronary vascular alterations (i.e., CMD and epicardial coronary spasm) can be accurately detected by performing an invasive functional assessment and are associated with adverse outcomes in both INOCA and MINOCA patients. Furthermore, the implementation of a tailored patient management demonstrated to improve patient’s symptoms and prognosis. However, the limited knowledge of myocardial ischaemia with non-obstructive coronary arteries precludes specific therapeutic interventions and, therefore, further research is warranted aiming to elucidate the underlying mechanisms and risk factors and to develop personalized forms of treatment.

## Abbreviations

IHD, Ischemic Heart Disease; OCAD, Obstructive Coronary Artery Disease; CMD, Coronary Microvascular Dysfunction; MI, Myocardial Infarction; INOCA, Ischemia with Non-Obstructive Coronary Arteries; MINOCA, Myocardial Infarction with non-Obstructive Coronary Arteries; VSA, Vasospastic Angina; VSMCs, Vascular Smooth Muscle Cells; CBF, Coronary Blood Flow; MVA, Microvascular Angina; NO, Nitric Oxide; TIMI, Thrombolysis In Myocardial Infarction; CFR, Coronary Flow Reserve; IMR, Index of Microvascular Resistances; HMR, Hyperaemic Microvascular Resistance; ACh, Acetylcholine; ECG, Electrocardiographic; MACE, Major Adverse Cardiovascular Events; CCBs, Calcium-Channel Blockers; ACEi, Angiotensin Converting Enzyme inhibitors; ARBs, Angiotensin-II Receptor Blockers; cGMP, cyclic Guanosine Monophosphate; DAPT, Dual Antiplatelet Therapy; MB, Myocardial Bridging.

## References

[1] Roth GA, Mensah GA, Johnson CO, Addolorato G, Ammirati E, Baddour LM (2020). Global Burden of Cardiovascular Diseases and Risk Factors, 1990–2019: Update from the GBD 2019 Study. *Journal of the American College of Cardiology*.

[2] Ibanez B, James S, Agewall S, Antunes MJ, Bucciarelli-Ducci C, Bueno H (2018). 2017 ESC Guidelines for the management of acute myocardial infarction in patients presenting with ST-segment elevation: The Task Force for the management of acute myocardial infarction in patients presenting with ST-segment elevation of the European Society of Cardiology (ESC). *European Heart Journal*.

[3] Knuuti J, Wijns W, Saraste A, Capodanno D, Barbato E, Funck-Brentano C (2020). 2019 ESC Guidelines for the diagnosis and management of chronic coronary syndromes. *European Heart Journal*.

[4] Collet JP, Thiele H, Barbato E, Barthélémy O, Bauersachs J, Bhatt DL (2021). 2020 ESC Guidelines for the management of acute coronary syndromes in patients presenting without persistent ST-segment elevation. *European Heart Journal*.

[5] Kaski JC, Crea F, Gersh BJ, Camici PG (2018). Reappraisal of Ischemic Heart Disease. *Circulation*.

[6] Pepine CJ, Anderson RD, Sharaf BL, Reis SE, Smith KM, Handberg EM (2010). Coronary microvascular reactivity to adenosine predicts adverse outcome in women evaluated for suspected ischemia results from the National Heart, Lung and Blood Institute WISE (Women’s Ischemia Syndrome Evaluation) study. *Journal of the American College of Cardiology*.

[7] Suwaidi JA, Hamasaki S, Higano ST, Nishimura RA, Holmes DR, Lerman A (2000). Long-term follow-up of patients with mild coronary artery disease and endothelial dysfunction. *Circulation*.

[8] Planer D, Mehran R, Ohman EM, White HD, Newman JD, Xu K (2014). Prognosis of Patients with Non–ST-Segment–Elevation Myocardial Infarction and Nonobstructive Coronary Artery Disease. *Circulation: Cardiovascular Interventions*.

[9] Montone RA, Niccoli G, Fracassi F, Russo M, Gurgoglione F, Cammà G (2018). Patients with acute myocardial infarction and non-obstructive coronary arteries: safety and prognostic relevance of invasive coronary provocative tests. *European Heart Journal*.

[10] Montone RA, Niccoli G, Russo M, Giaccari M, Del Buono MG, Meucci MC (2020). Clinical, angiographic and echocardiographic correlates of epicardial and microvascular spasm in patients with myocardial ischaemia and non-obstructive coronary arteries. *Clinical Research in Cardiology*.

[11] Niccoli G, Montone RA, Lanza GA, Crea F (2017). Angina after percutaneous coronary intervention: the need for precision medicine. *International Journal of Cardiology*.

[12] Gould KL, Lipscomb K, Hamilton GW (1974). Physiologic basis for assessing critical coronary stenosis. *The American Journal of Cardiology*.

[13] Beltrame JF, Crea F, Kaski JC, Ogawa H, Ong P, Sechtem U (2017). International standardization of diagnostic criteria for vasospastic angina. *European Heart Journal*.

[14] Camilli M, Russo M, Rinaldi R, Caffè A, La Vecchia G, Bonanni A (2022). Air Pollution and Coronary Vasomotor Disorders in Patients With Myocardial Ischemia and Unobstructed Coronary Arteries. *Journal of the American College of Cardiology*.

[15] Beltrame JF, Crea F, Camici P (2009). Advances in coronary microvascular dysfunction. *Heart, Lung and Circulation*.

[16] Padro T, Manfrini O, Bugiardini R, Canty J, Cenko E, De Luca G (2020). ESC Working Group on Coronary Pathophysiology and Microcirculation position paper on ‘coronary microvascular dysfunction in cardiovascular disease’. *Cardiovascular Research*.

[17] Taqueti VR, Di Carli MF (2018). Coronary Microvascular Disease Pathogenic Mechanisms and Therapeutic Options. *Journal of the American College of Cardiology*.

[18] Camici PG, Crea F (2007). Coronary Microvascular Dysfunction. *New England Journal of Medicine*.

[19] Crea F, Montone RA, Rinaldi R (2022). Pathophysiology of Coronary Microvascular Dysfunction. *Circulation Journal*.

[20] Crea F, Camici PG, Bairey Merz CN (2014). Coronary microvascular dysfunction: an update. *European Heart Journal*.

[21] Del Buono MG, Montone RA, Camilli M, Carbone S, Narula J, Lavie CJ (2021). Coronary Microvascular Dysfunction across the Spectrum of Cardiovascular Diseases. *Journal of the American College of Cardiology*.

[22] Montone RA, Iannaccone G, Meucci MC, Gurgoglione F, Niccoli G (2020). Myocardial and Microvascular Injury Due to Coronavirus Disease 2019. *European Cardiology*.

[23] Neumann FJ, Sousa-Uva M, Ahlsson A, Alfonso F, Banning AP, Benedetto U (2019). 2018 ESC/EACTS Guidelines on myocardial revascularization. *European Heart Journal*.

[24] Montone RA, Galiuto L, Meucci MC, Del Buono MG, Vergni F, Camilli M (2020). Coronary slow flow is associated with a worse clinical outcome in patients with Takotsubo syndrome. *Heart*.

[25] Ong P, Camici PG, Beltrame JF, Crea F, Shimokawa H, Sechtem U (2018). International standardization of diagnostic criteria for microvascular angina. *International Journal of Cardiology*.

[26] Ong P, Safdar B, Seitz A, Hubert A, Beltrame JF, Prescott E (2020). Diagnosis of coronary microvascular dysfunction in the clinic. *Cardiovascular Research*.

[27] Perera D, Berry C, Hoole SP, Sinha A, Rahman H, Morris PD (2022). Invasive coronary physiology in patients with angina and non-obstructive coronary artery disease: a consensus document from the coronary microvascular dysfunction workstream of the British Heart Foundation/National Institute for Health Research Partnership. *Heart*.

[28] Rahman H, Demir OM, Ryan M, McConkey H, Scannell C, Ellis H (2020). Optimal Use of Vasodilators for Diagnosis of Microvascular Angina in the Cardiac Catheterization Laboratory. *Circulation: Cardiovascular Interventions*.

[29] AlBadri A, Bairey Merz CN, Johnson BD, Wei J, Mehta PK, Cook-Wiens G (2019). Impact of Abnormal Coronary Reactivity on Long-Term Clinical Outcomes in Women. *Journal of the American College of Cardiology*.

[30] Lee JM, Jung J, Hwang D, Park J, Fan Y, Na S (2016). Coronary Flow Reserve and Microcirculatory Resistance in Patients with Intermediate Coronary Stenosis. *Journal of the American College of Cardiology*.

[31] Kelshiker MA, Seligman H, Howard JP, Rahman H, Foley M, Nowbar AN (2022). Coronary flow reserve and cardiovascular outcomes: a systematic review and meta-analysis. *European Heart Journal*.

[32] Xaplanteris P, Fournier S, Keulards DCJ, Adjedj J, Ciccarelli G, Milkas A (2018). Catheter-Based Measurements of Absolute Coronary Blood Flow and Microvascular Resistance. *Circulation: Cardiovascular Interventions*.

[33] Fearon WF, Balsam LB, Farouque HMO, Robbins RC, Fitzgerald PJ, Yock PG (2003). Novel Index for Invasively Assessing the Coronary Microcirculation. *Circulation*.

[34] Ng MK, Yeung AC, Fearon WF (2006). Invasive assessment of the coronary microcirculation: superior reproducibility and less hemodynamic dependence of index of microcirculatory resistance compared with coronary flow reserve. *Circulation*.

[35] Williams RP, de Waard GA, De Silva K, Lumley M, Asrress K, Arri S (2018). Doppler Versus Thermodilution-Derived Coronary Microvascular Resistance to Predict Coronary Microvascular Dysfunction in Patients with Acute Myocardial Infarction or Stable Angina Pectoris. *The American Journal of Cardiology*.

[36] Kunadian V, Chieffo A, Camici PG, Berry C, Escaned J, Maas AHEM (2020). An EAPCI Expert Consensus Document on Ischaemia with Non-Obstructive Coronary Arteries in Collaboration with European Society of Cardiology Working Group on Coronary Pathophysiology & Microcirculation Endorsed by Coronary Vasomotor Disorders International Study Group. *European Heart Journal*.

[37] Montone RA, Meucci MC, De Vita A, Lanza GA, Niccoli G (2021). Coronary provocative tests in the catheterization laboratory: Pathophysiological bases, methodological considerations and clinical implications. *Atherosclerosis*.

[38] Zaya M, Mehta PK, Bairey Merz CN (2014). Provocative Testing for Coronary Reactivity and Spasm. *Journal of the American College of Cardiology*.

[39] Montone RA, Rinaldi R, Del Buono MG, Gurgoglione F, La Vecchia G, Russo M (2022). Safety and prognostic relevance of acetylcholine testing in patients with stable myocardial ischaemia or myocardial infarction and non-obstructive coronary arteries. *EuroIntervention*.

[40] Hackett D, Larkin S, Chierchia S, Davies G, Kaski JC, Maseri A (1987). Induction of coronary artery spasm by a direct local action of ergonovine. *Circulation*.

[41] Ford TJ, Stanley B, Good R, Rocchiccioli P, McEntegart M, Watkins S (2018). Stratified Medical Therapy Using Invasive Coronary Function Testing in Angina. *Journal of the American College of Cardiology*.

[42] Kosugi M, Nakagomi A, Shibui T, Kato K, Kusama Y, Atarashi H (2011). Effect of Long-Term Nitrate Treatment on Cardiac Events in Patients with Vasospastic Angina. *Circulation Journal*.

[43] Russo G, Di Franco A, Lamendola P, Tarzia P, Nerla R, Stazi A (2013). Lack of effect of nitrates on exercise stress test results in patients with microvascular angina. *Cardiovascular Drugs and Therapy*.

[44] Neglia D, Fommei E, Varela-Carver A, Mancini M, Ghione S, Lombardi M (2011). Perindopril and indapamide reverse coronary microvascular remodelling and improve flow in arterial hypertension. *Journal of Hypertension*.

[45] Pauly DF, Johnson BD, Anderson RD, Handberg EM, Smith KM, Cooper-DeHoff RM (2011). In women with symptoms of cardiac ischemia, nonobstructive coronary arteries, and microvascular dysfunction, angiotensin-converting enzyme inhibition is associated with improved microvascular function: a double-blind randomized study from the National Heart, Lung and Blood Institute Women’s Ischemia Syndrome Evaluation (WISE). *American Heart Journal*.

[46] Robertson RM, Wood AJ, Vaughn WK, Robertson D (1982). Exacerbation of vasotonic angina pectoris by propranolol. *Circulation*.

[47] Ishii M, Kaikita K, Sato K, Yamanaga K, Miyazaki T, Akasaka T (2016). Impact of Statin Therapy on Clinical Outcome in Patients with Coronary Spasm. *Journal of the American Heart Association*.

[48] Jaw-Wen C, Wen-Lieng L, Nai-Wei H, Shing-Jong L, Chih-Tai T, Shih-Pu W (1997). Effects of short-term treatment of nicorandil on exercise-induced myocardial ischemia and abnormal cardiac autonomic activity in microvascular angina. *The American Journal of Cardiology*.

[49] Bairey Merz CN, Handberg EM, Shufelt CL, Mehta PK, Minissian MB, Wei J (2016). A randomized, placebo-controlled trial of late Na current inhibition (ranolazine) in coronary microvascular dysfunction (CMD): impact on angina and myocardial perfusion reserve. *European Heart Journal*.

[50] Villano A, Di Franco A, Nerla R, Sestito A, Tarzia P, Lamendola P (2013). Effects of Ivabradine and Ranolazine in Patients with Microvascular Angina Pectoris. *The American Journal of Cardiology*.

[51] Masumoto A, Mohri M, Shimokawa H, Urakami L, Usui M, Takeshita A (2002). Suppression of Coronary Artery Spasm by the Rho-Kinase Inhibitor Fasudil in Patients with Vasospastic Angina. *Circulation*.

[52] Mohri M, Shimokawa H, Hirakawa Y, Masumoto A, Takeshita A (2003). Rho-kinase inhibition with intracoronary fasudil prevents myocardial ischemia in patients with coronary microvascular spasm. *Journal of the American College of Cardiology*.

[53] Suda A, Takahashi J, Hao K, Kikuchi Y, Shindo T, Ikeda S (2019). Coronary Functional Abnormalities in Patients with Angina and Nonobstructive Coronary Artery Disease. *Journal of the American College of Cardiology*.

[54] Sorop O, van den Heuvel M, van Ditzhuijzen NS, de Beer VJ, Heinonen I, van Duin RWB (2016). Coronary microvascular dysfunction after long-term diabetes and hypercholesterolemia. *American Journal of Physiology-Heart and Circulatory Physiology*.

[55] Handberg EM, Merz CNB, Cooper-Dehoff RM, Wei J, Conlon M, Lo MC (2021). Rationale and design of the Women’s Ischemia Trial to Reduce Events in Nonobstructive CAD (WARRIOR) trial. *American Heart Journal*.

[56] Jespersen L, Hvelplund A, Abildstrom SZ, Pedersen F, Galatius S, Madsen JK (2012). Stable angina pectoris with no obstructive coronary artery disease is associated with increased risks of major adverse cardiovascular events. *European Heart Journal*.

[57] Rahman H, Demir OM, Khan F, Ryan M, Ellis H, Mills MT (2020). Physiological Stratification of Patients with Angina Due to Coronary Microvascular Dysfunction. *Journal of the American College of Cardiology*.

[58] Maron DJ, Hochman JS, Reynolds HR, Bangalore S, O’Brien SM, Boden WE (2020). Initial Invasive or Conservative Strategy for Stable Coronary Disease. *The New England Journal of Medicine*.

[59] Niccoli G, Scalone G, Crea F (2015). Acute myocardial infarction with no obstructive coronary atherosclerosis: mechanisms and management. *European Heart Journal*.

[60] Montone RA, Jang I, Beltrame JF, Sicari R, Meucci MC, Bode M (2021). The evolving role of cardiac imaging in patients with myocardial infarction and non-obstructive coronary arteries. *Progress in Cardiovascular Diseases*.

[61] Montone RA, Niccoli G, Crea F, Jang I (2020). Management of non-culprit coronary plaques in patients with acute coronary syndrome. *European Heart Journal*.

[62] Crea F, Niccoli G (2019). Myocardial Infarction with Nonobstructive Coronary Atherosclerosis. *JACC: Cardiovascular Imaging*.

[63] Nordenskjöld AM, Agewall S, Atar D, Baron T, Beltrame J, Bergström O (2021). Randomized evaluation of beta blocker and ACE-inhibitor/angiotensin receptor blocker treatment in patients with myocardial infarction with non-obstructive coronary arteries (MINOCA-BAT): Rationale and design. *American Heart Journal*.

[64] Lindahl B, Baron T, Erlinge D, Hadziosmanovic N, Nordenskjöld A, Gard A (2017). Medical Therapy for Secondary Prevention and Long-Term Outcome in Patients with Myocardial Infarction with Nonobstructive Coronary Artery Disease. *Circulation*.

[65] Pasupathy S, Air T, Dreyer RP, Tavella R, Beltrame JF (2015). Systematic review of patients presenting with suspected myocardial infarction and nonobstructive coronary arteries. *Circulation*.

[66] Del Buono MG, Montone RA, Iannaccone G, Meucci MC, Rinaldi R, D’Amario D (2021). Diagnostic work-up and therapeutic implications in MINOCA: need for a personalized approach. *Future Cardiology*.

[67] Del Buono MG, La Vecchia G, Rinaldi R, Sanna T, Crea F, Montone RA (2022). Myocardial infarction with nonobstructive coronary arteries: the need for precision medicine. *Current Opinion in Cardiology*.

[68] Montone RA, Cosentino N, Graziani F, Gorla R, Del Buono MG, La Vecchia G (2022). Precision medicine versus standard of care for patients with myocardial infarction with non-obstructive coronary arteries (MINOCA): rationale and design of the multicentre, randomised PROMISE trial. *EuroIntervention*.

[69] Sternheim D, Power DA, Samtani R, Kini A, Fuster V, Sharma S (2021). Myocardial Bridging: Diagnosis, Functional Assessment, and Management. *Journal of the American College of Cardiology*.

[70] Matta A, Nader V, Canitrot R, Delmas C, Bouisset F, Lhermusier T (2022). Myocardial bridging is significantly associated to myocardial infarction with non-obstructive coronary arteries. *European Heart Journal. Acute Cardiovascular Care*.

[71] Montone RA, Gurgoglione FL, Del Buono MG, Rinaldi R, Meucci MC, Iannaccone G (2021). Interplay Between Myocardial Bridging and Coronary Spasm in Patients With Myocardial Ischemia and Non-Obstructive Coronary Arteries: Pathogenic and Prognostic Implications. *Journal of the American Heart Association*.

